# An obituary of Dr. Ding-Shinn Chen

**DOI:** 10.1186/s12929-020-00675-6

**Published:** 2020-07-30

**Authors:** JBS Editorial Office

**Affiliations:** Editorial Office of Journal of Biomedical Science, Taipei Medical University, 250 Wu-Hsing Street, Taipei, 110 Taiwan

**Figure 1:**
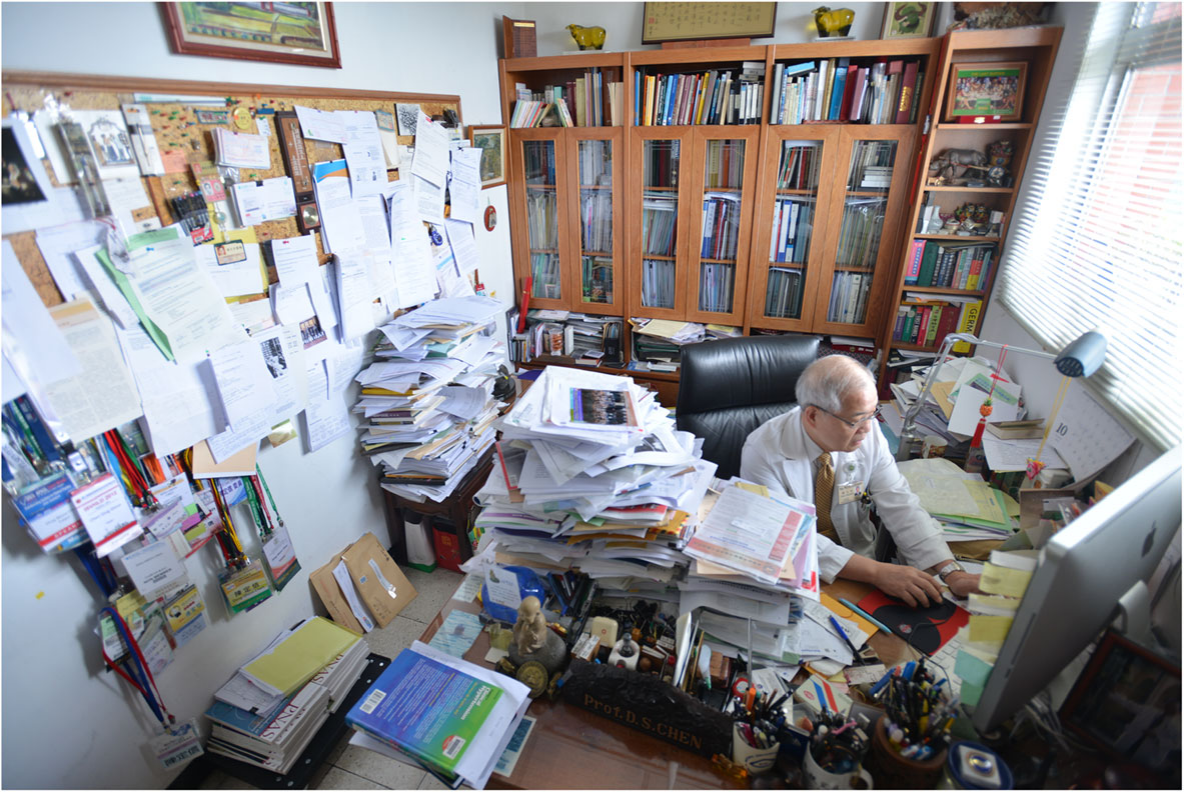
Dr. Chen is known to be a very hard-working person. He likes to compare himself to the domesticated water buffalo in Taiwan, which are very common in the farmers’ rice field during his childhood. In his office, he collected many paintings, sculptures, and photos of water buffalo. It is clear that he is a believer in the virtue of hard-working quietly without complaints.

Professor Ding-Shinn Chen, an internationally renowned hepatologist, passed away on Wednesday, June 24, 2020. He died from pancreatic cancer at National Taiwan University Hospital (NTUH) at age 77. As a highly accomplished physician scientist, Dr. Chen is also known as a great educator, a humorous lecturer, a caring medical doctor, a skillful administrator, a well-liked colleague, and a warm friend to the academic and scientific community, particularly to the research field of viral hepatitis.

Dr. Chen is one of the founding Editors of our Journal of Biomedical Science. Despite his extremely busy daily activities in clinics, teaching, research, and administration, he had served continuously as an associate editor until the very last day, when he bid his farewell to all of us. Over the past 25 years, he witnessed the long and winding road of this Journal’s growth and struggle - from impact factor (IF) zero to being enlisted by JCR (IF 0.99), to the most recent IF 5.762 in 2019. His death is truly a big loss to our Journal and to the entire medical and scientific community, in Taiwan and worldwide!

Dr. Chen was born in the Ying-Ge Township, New Taipei City, Taiwan, in 1943. He graduated from National Taiwan University, College of Medicine, in 1968, and received medical residency training at NTUH in 1969–1973. Professor Sung Juei-low at NTUH is considered the father of liver disease research in Taiwan. In 1973, Dr. Chen chose to specialize in gastroenterology, and made the best decision in his career by joining Professor Sung’s research team. He was involved in measuring the prevalence rate of hepatitis B virus (HBV) surface antigen (HBsAg) in liver disease patients. In 1975, Dr. Chen became an instructor at NTU. Arranged by Professor Sung, for four months in 1974–1975, he learned how to use new methods to detect HBsAg, and performed its serotyping, in the laboratory of Dr. Kusuya Nishioka at National Cancer Center Research Institute in Tokyo, Japan. Later in 1979–1980, he was a visiting scientist in Dr. Robert H. Purcell’s laboratory at NIH in Bethesda, USA. In this one-year period, he was first exposed to molecular biology by studying HBV DNA integration in hepatomas.

Professor Sung and his protégé DS Chen demonstrated that the prevalence rate of HBsAg in Taiwan could be as high as 18%. They also noted that one common mode of virus transmission is the mother-to-infant transmission during delivery. Soon after his return from NIH to NTUH, Taiwan, in 1981, Chen and Sung would like to test if the HBV vaccine could be effective in reducing the incidence of viral hepatitis and liver diseases in Taiwan. The timing was indeed perfect, when Dr. Palmer Beasley, in close collaborations with Professor Chin-Yun Lee and several hospitals in Taipei, reported in *the Lancet* (1983) the efficacies of hepatitis B immune globulin (HBIG) in blocking maternal-infant infection, as well as hepatitis B vaccine in its antibody production. HBV vaccine appeared to be safe for immunization of Taiwanese infants. Sung and Chen were then serving on a government consultation team. They advocated actively the vaccine idea to the society and Taiwan government, leading to a nationwide vaccination program for the newborn babies in Taiwan in 1984. Ms. Hsu-Mei Hsu, a card-carrying epidemiologist in the government sector, who happens to be Dr. Chen’s wife, also played a key role in the entire planning and successful execution of this bold and unprecedented nationwide vaccination program in history! According to a classic paper published in 1997 in the *New England Journal of Medicine* (first authored by professor Mei-Hwei Chang and last authored by DS Chen), one decade after launching the vaccination program, the carrier rate in the vaccinated population dropped from 15% to less than 1%. Furthermore, the liver cancer incidence in children also decreased significantly. This is the first successful example of a human cancer vaccine!

In addition to hepatitis B, Dr. Chen and his colleagues also made significant contributions to the treatment of hepatitis C. For example, Drs. Ming-Yang Lai and Chen succeeded in treating hepatitis C patients with the combination of alpha-interferon and ribavirin. Back in the earlier days, this combination therapy was the best approach for treatment of hepatitis C.

In Dr. Chen’s illustrious career, he received numerous prestigious awards and recognitions. For example, in 1992, he was elected to be an Academician, Academia Sinica, Taiwan. In 2002, he received an honorary degree from Kaohsiung Medical University, the best medical school in Southern Taiwan. In 2005, he was elected as a member of the US National Academy of Sciences, a rare honor bestowed to few foreign associates. In 2006, he received the Trieste Science Prize from Third World Academy of Sciences (TWAS). In 2007, he was the recipient of the Presidential Award in Taiwan. In 2009, EASL International Recognition Award. In 2010, Japan Nikkei Asia Prize. In 2011, he was honored with a Distinguished Clinician Educator/Mentor Award from AASLD (American Association for the Study of Liver Diseases). In 2014, Fellow of AASLD. In 2016, EASL Hall of Fame. In 2018, the Blumberg Award from the Hepatitis B Foundation, USA. Dr. Baruch Blumberg won the Nobel Prize for his discovery of hepatitis B virus. As of January, 2019, Dr. Chen published a total of 750 original articles, 44 book chapters, and edited 3 books.

Dr. Chen is also known as a talented administrator. For example, in 2001–2007, he served as the Dean of National Taiwan University, College of Medicine. For the sake of a more concise obituary, we will not go into details of his many important positions in the government, university, hospital, and professional societies. In appreciation of his many important contributions to clinical medicine and the national healthcare policy, Dr. Chen was honored with a 2nd-ranked Jin-Hsing Medal from the current Taiwan President Tsai Ing-wen in 2018.

Dr. Chen is known to be a very hard-working person. He likes to compare himself to the domesticated water buffalo in Taiwan, which are very common in the farmers’ rice field during his childhood. In his office, he collected many paintings, sculptures, and photos of water buffalo. It is clear that he is a believer in the virtue of hard-working quietly without complaints.

Although Dr. Chen officially retired from NTU in 2013, he continued to be active in liver research and teaching young doctors in the NTUH hospital. Dr. Chen is survived by his wife Hsu-Mei Hsu – an epidemiologist; his son Chih-Heng (Henry) Chen - in the finance field, and his daughter Yun-Ru (Ruby) Chen – a protein chemist in Academia Sinica, Taiwan. Last, but not the least, Dr. Chen is also survived by many of his students and trainees, who have been active and successful in liver disease research. There is no doubt that the legend of Dr. Ding-Shinn Chen and his Taiwan buffalo spirit will continue to live on in generations to come!

A public memorial service for Dr. Ding-Shinn Chen will be held at National Taiwan University, College of Medicine, Lecture Hall, on August 16, 2020.

